# Inefficacy of Repetitive Transcranial Magnetic Stimulation in Parkinson’s Disease Patients with Levodopa-Induced Dyskinesias: Results from a Pilot Study

**DOI:** 10.3390/biomedicines13071663

**Published:** 2025-07-08

**Authors:** Alma Medrano-Hernández, Gabriel Neri-Nani, Mayela Rodríguez-Violante, René Drucker-Colín, Anahí Chavarría

**Affiliations:** 1 Unidad de Medicina Experimental “Ruy Pérez Tamayo”, Facultad de Medicina, Universidad Nacional Autónoma de México, Mexico City CP 06726, Mexico; dra.alma.medrano@gmail.com; 2 Programa de Doctorado en Ciencias Biomédicas, Universidad Nacional Autónoma de México, Mexico City CP 04510, Mexico; 3 Unidad de Trastornos del Movimiento y Sueño, Hospital de General Ajusco Medio, Secretaría de Salud de la Ciudad de México, Mexico City CP 14250, Mexico; 4 Laboratorio Clínico de Enfermedades Neurodegenerativas, Instituto Nacional de Neurología y Neurocirugía Manuel Velasco Suárez, Secretaría de Salud, Mexico City CP 14269, Mexico; mrodriguez@innn.edu.mx; 5 Departamento de Neuropatología Molecular, Instituto de Fisiología Celular, Universidad Nacional Autónoma de México, Mexico City CP 04510, Mexico

**Keywords:** Parkinson’s disease, levodopa-induced dyskinesia, repetitive transcranial magnetic stimulation

## Abstract

**Background:** Parkinson’s disease (PD) presents a significant challenge due to its wide range of motor, non-motor, and treatment-related symptoms. Non-invasive interventions like transcranial magnetic stimulation (TMS) are being explored for potential therapeutic benefits. This study aimed to assess if a high-frequency repetitive TMS protocol (HF-rTMS) consisting of 10 trains of 100 pulses of rTMS at 25 Hz over the motor cortex (M1) at 80% of the resting motor threshold could be effective in treating motor or non-motor symptoms in patients with PD with levodopa-induced dyskinesias. **Methods:** A randomized, single-blinded, placebo-controlled pilot trial was conducted with eleven PD patients. Nine patients received HF-rTMS, while two received sham stimulation. Patients were exhaustively evaluated using validated clinical scales to assess motor and non-motor symptoms. The study followed a rigorous protocol to avoid bias, with assessments conducted by a neurologist specialized in single-blinded movement disorder. **Results:** The HF-rTMS group experienced a statistically significant slight worsening in both motor and non-motor symptoms, particularly in the mood/cognition and gastrointestinal domains. However, positive effects were observed in some non-motor symptoms, specifically reduced excessive sweating and weight. No adverse effects were reported. **Conclusions**: Although HF-rTMS did not produce significant motor improvements, its potential benefit on specific non-motor symptoms, such as autonomic regulation, warrants further investigation.

## 1. Introduction

Parkinson’s disease (PD) stands as a neurodegenerative disorder with a calculated incidence of 14.9 per 100,000 Mexicans [1]. Although various pharmacological treatment options are available [2], levodopa is more commonly used in Mexico than in other countries, as 75% of PD patients use it during disease management [3]. The adverse motor effects of levodopa include motor fluctuations and levodopa-induced dyskinesias (LID). Within the first year of treatment, about 40% of patients will exhibit LID, which occurs more frequently in those with severe PD who need larger levodopa doses [4]. LID diminishes the overall quality of life, particularly in the activities of daily living, emotional well-being, cognition, and communication domains [5,6]. The management of advanced PD with motor fluctuations and LID presents a clinical conundrum; even though levodopa adjustments and pharmacological treatment with amantadine are effective options, the adverse effects in terms of neurological and non-neurological manifestations have encouraged the development of other treatments [5,6,7,8,9,10]. Deep brain stimulation (DBS) has been reported to reduce dyskinesia within the globus pallidus internus or subthalamic nucleus (STN) stimulation by around 89% and 62%, respectively [7,9,10]. Several studies suggest that the anti-dyskinetic effect of STN DBS is more dependent on medication reduction than on a direct anti-dyskinetic effect [6,10]. Unfortunately, some patients are not suitable for invasive treatments or do not have access to them due to the high costs they represent, so there is a need for effective, non-invasive therapeutic options that can manage LID and motor fluctuations in PD.

Electrical and magnetic stimulation of the human brain can be used to activate or suppress neurons. Transcranial magnetic stimulation (TMS) is a non-invasive neuromodulation method that uses a magnetic coil placed over the scalp to generate an electric field, influencing ongoing brain activity [11,12,13,14]. After placing the magnetic coil, a short, high-intensity magnetic field pulse can be produced, creating electric currents strong enough to cause neuron depolarization [15]. The stimulation of the primary motor cortex (M1) activates the corticospinal tract, inducing contraction in the corresponding contralateral muscle. These contractions can be recorded and are called motor-evoked potentials (MEPs) [16]. Various frequencies of repetitive transcranial magnetic stimulation (rTMS) can be programmed to modulate neural activity. The effects on the brain can last minutes to hours after the stimulation has ended [17,18,19].

rTMS partially enhances functionality by modulating neural connectivity and exerting local and distant effects through connectivity between regions, which can be revealed behaviorally, physiologically, or by combining TMS with neuroimaging [20,21,22,23]. Animal models have shown that rTMS effects rely on NMDA receptor-mediated glutamatergic function, suggesting the involvement of long-term potentiation and depression mechanisms [24]. Furthermore, other processes, such as neurotrophic, neuroinflammatory, and neuroendocrine factors, as well as the neuroglia network, appear to contribute to the observable after-effects [25].

rTMS can be classified as low-frequency (1 Hz or less) rTMS (LF-rTMS), which is assumed to decrease cortical excitability. High-frequency (5 Hz or greater) rTMS (HF-rTMS) appears to result in a persistent augmentation of motor-evoked size along with a concomitant reduction in cortical inhibition activity [11]. This assumption is not the only variable defining the effect on cortical function, as the inter-train interval in a high-frequency rTMS protocol and the number of pulses given can vary the long-term effects [26,27,28].

rTMS has been studied in different diseases; in PD, it has been shown that stimulating M1 bilaterally using HF-rTMS can be recommended for treating motor symptoms. Various protocols have been used, but intensified designs using a more significant number of sessions (even including more sessions per day) could be a way to optimize the efficacy of rTMS [29]. Alternatively, non-invasive TMS of M1 could be combined with invasive deep brain stimulation to promote associative plasticity in the brain circuits of motor control [29].

In our previous study, we applied 25 Hz rTMS over M1 in a 12-week protocol, yielding positive results in improving motor symptoms in patients with PD, 40% of whom also exhibited LIDs [30]. However, the LIDs were not studied in detail. Given the short-term positive effects observed, extending the duration of the same rTMS protocol could further improve motor symptoms, potentially allowing for a reduction in levodopa dosage. This study aimed to assess whether using the same intensity protocol over an extended period could benefit PD patients with LIDs, focusing on the potential for levodopa dosage reduction or its application as a targeted treatment option for LIDs.

## 2. Materials and Methods

### 2.1. Subjects

**Figure 1:**
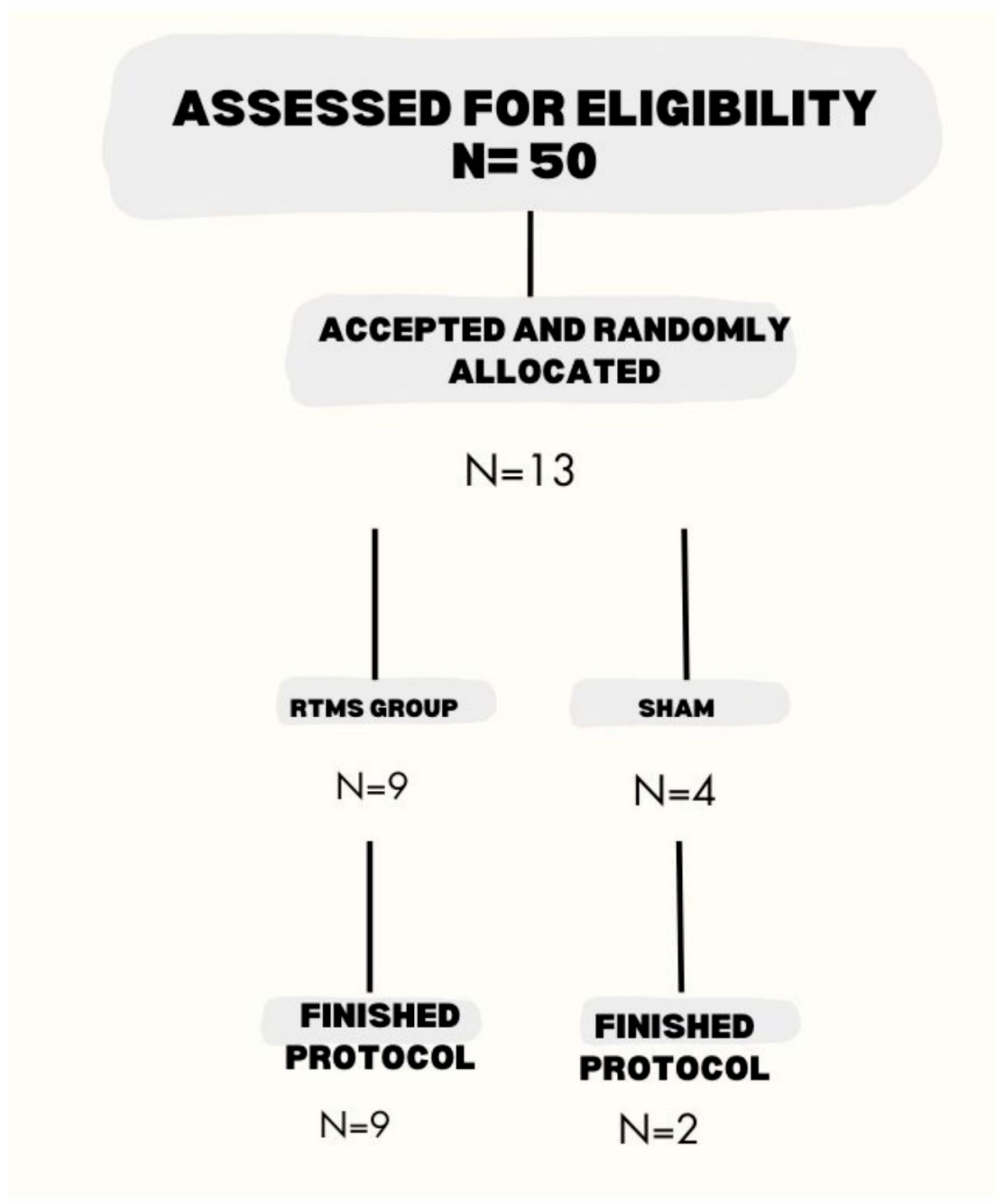
Patient disposition. Of the 50 eligible patients, only 13 signed the informed consent and were scheduled for the first evaluation. After the initial assessment, two patients in the sham arm left the study because of medical conditions other than PD. Nine patients finished the rTMS and two finished the sham protocol.

Thirteen patients with idiopathic PD (Hoehn and Yahr Scale 2–3 in off-state), according to the diagnostic criteria of the UK Parkinson’s Disease Society Brain Bank [12], participated in the study (Figure 1). Clinical details of the patients are shown in Table 1. Nine patients were randomly assigned to the experimental patient (EP) group and the other two to the sham patient group (SP). The study obtained the approval of the ethics committee of the Secretaría de Salud de la Ciudad de México, registration number 211/101/999/16. Written informed consent was obtained from all participants. The study was conducted from August 2015 to September 2017 and followed the principles of Good Clinical Practice and the Declaration of Helsinki.

### 2.2. Inclusion Criteria

The main inclusion criteria were: ≥18 years old; diagnosis of PD according to the UK Parkinson’s Disease Society Brain Bank criteria and motor complications associated with levodopa treatment, both assessed by certificated movement disorder neurologists; levodopa on a stable dose ≥600 mg/day (in combination with benserazide or carbidopa) for at least 28 days before baseline; and a Mini-Mental State Examination score ≥25. Patients were on fixed doses of their usual anti-Parkinsonian medication for at least one month before starting the protocol until the end of the study. Amantadine was permitted if the patient was on stable doses for at least 28 days before baseline and was expected to be maintained for the duration of the study. The exclusion criteria primarily included conditions where rTMS or previous treatments involving this technique were not applicable.

### 2.3. Procedure

**Figure 2:**
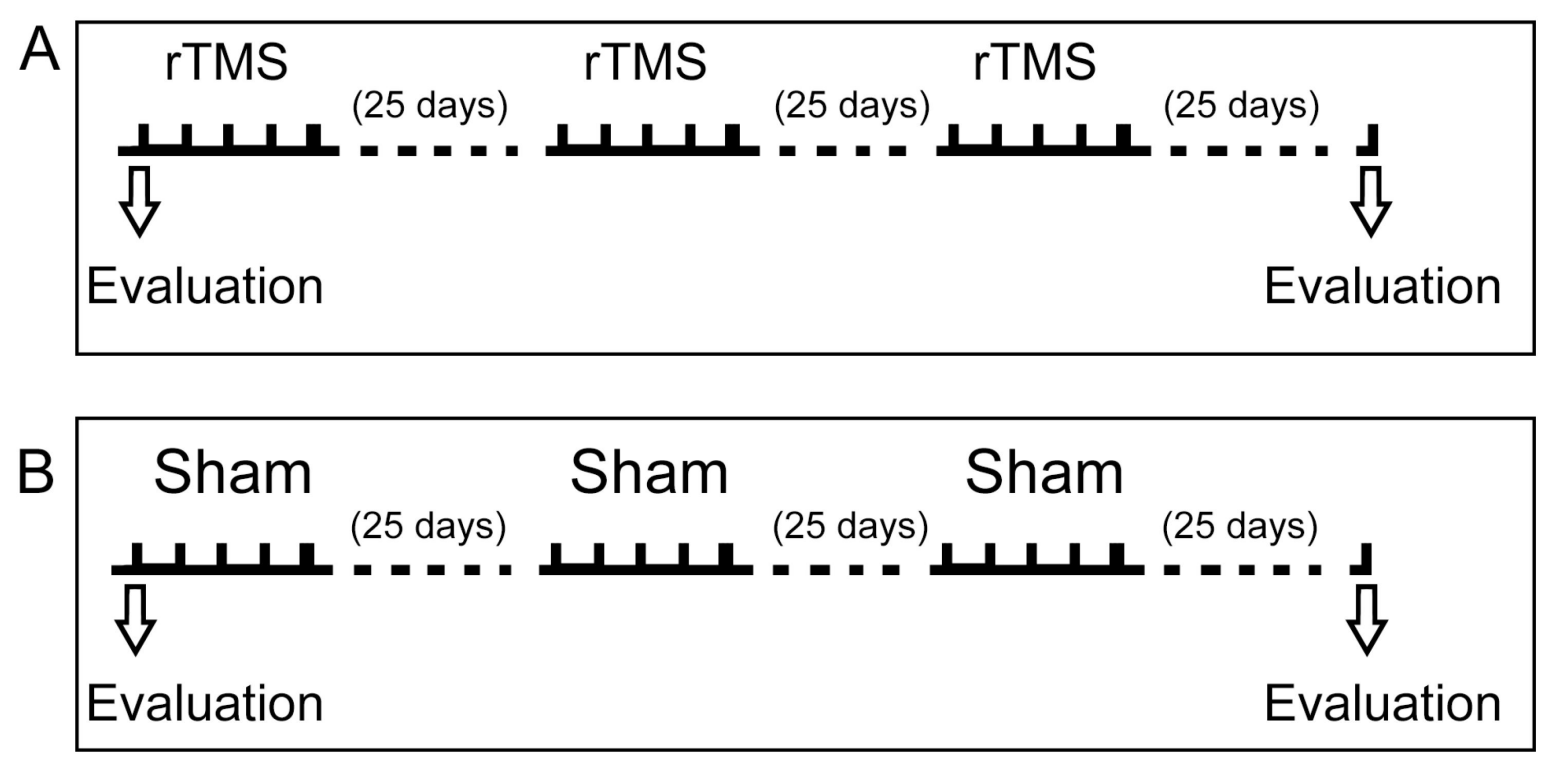
General treatment schedule. (**A**) Baseline evaluation was conducted in off-state and before the rTMS application. Patients were evaluated during the morning before rTMS using the Movement Disorder Society Unified Parkinson’s Disease Rating Scale, the Unified Dyskinesia Rating Scale, and the Non-Motor Symptom Assessment Scale for Parkinson’s Disease. The rTMS protocol is detailed in the methodology. (**B**) The same evaluation considerations were applied to the sham group.

The overall experimental procedure consisted of three rTMS sessions with clinical evaluation before and after the rTMS treatment. Figure 2 shows a scheme of the various stages and their timings, with their details described below.

### 2.4. Transcranial Magnetic Stimulation

Stimulation was administered to patients 60 min after their medication intake, with all sessions conducted in the morning at the neurology service of Ajusco Medio Hospital by the same trained physician. The room temperature was maintained at 25 °C. During the experiment, patients were seated in a quiet environment. At the same time, motor cortex excitability was assessed using a Rapid2 Magstim^®^ device (Magstim Co., Ltd., Whitland, Southwest Wales, UK) equipped with an 80 mm figure-of-eight coil. The coil was positioned over M1, explicitly targeting the right abductor pollicis brevis (APB) muscle, with a mechanical arm ensuring the coil remained tangential to the scalp at a 45° angle. MEPs were recorded using electromyography.

Transcranial magnetic stimulation (TMS) intensity was individualized based on the resting motor threshold (RMT), defined as the minimum stimulus intensity required to evoke an MEP of ≥50 μV in at least 5 out of 10 consecutive trials of the relaxed muscle. The repetitive TMS (rTMS) protocol involved bilateral stimulation, comprising 10 trains of 100 pulses delivered at 25 Hz over M1 at 80% of the RMT for the right APB muscle. The experimental group (EP) received active stimulation, while in the SP, the coil was positioned vertically to prevent stimulation while replicating the sensation of coil placement. This 15-session protocol was implemented five times during the first week of each month for three months. Patients were evaluated during different weeks to prevent communication between them.

### 2.5. Clinical Evaluation

To ensure consistency, a movement disorder specialist neurologist, blinded to the procedure, evaluated the patients in the off-medication state one hour before the rTMS session. The assessments were conducted using the Movement Disorder Society Unified Parkinson’s Disease Rating Scale (MDS-UPDRS), the Unified Dyskinesia Rating Scale (UDysRS), and the Non-Motor Symptom Assessment Scale for Parkinson’s Disease (NMS) (Figure 2). No changes were made to the patients’ Parkinson’s disease treatment regimen throughout the study.

### 2.6. Study Design

This study used a randomized, single-blind, placebo-controlled, two-arm design to evaluate the long-term effects of HF-rTMS on motor and non-motor symptoms in PD and LIDs. Evaluations were conducted by a single-blinded movement disorder neurologist, ensuring the consistency of the assessments. Patients were evaluated in the off-medication state using the MDS-UPDRS, the UDysRS, and the NMS. To eliminate potential information bias, the sham and stimulation groups were assessed in separate weeks, preventing participant interaction. HF-rTMS administration was standardized, with all sessions conducted by the same physician trained in brain stimulation techniques.

### 2.7. Statistical Analysis

Statistical analyses were performed using the Statistical Package for the Social Sciences (SPSS), version 25. The normal distribution of the variables was assessed using the Kolmogorov–Smirnov test. For normally distributed variables, the Fisher test was applied. Differences were analyzed using the non-parametric Mann–Whitney U and Wilcoxon tests for variables that did not meet normal distribution criteria. The data are presented as median ±25–75 centile and statistical significance was set at *p* < 0.05.

## 3. Results

### 3.1. Patient Characteristics

Thirteen right-handed patients were randomly assigned: nine to the rTMS group and four to the sham group. Two patients in the sham group left the study because of medical conditions other than PD. Patient disposition is shown in Figure 1. Patients in the rTMS group were older than the sham patients. Disease duration was longer in the rTMS group; in both groups, initial PD manifestations were in the left hand. There was no difference between the two groups in the treatment scheme and dose for PD and dyskinesias. None received treatment with monoamine oxidase inhibitors or anticholinergic inhibitors. None had a family history of PD or tremor (Table 1).

### 3.2. Clinical Evaluation Showed No Differences Between Groups Before and After Intervention

The baseline evaluation between both groups using the MDS-UPDRS, the Hoehn and Yahr Scale (H&Y), the Non-Motor Symptom Assessment Scale for PD (NMS), and the Unified Dyskinesia Rating Scale (UDysRS) presented no statistical difference. The only evaluation with a statistical tendency was the MDS-UPDRS Part 3 scale (*p* = 0.059), which was more affected than the sham group (Table 2).

The MDS-UPDRS evaluation showed no differences between parts 1 and 2. The rTMS group worsened in parts 3 and 4; the analysis performed on the different components that conformed to the scale showed that left-hand, posture, and left-hand kinetic tremors were the most affected post-rTMS treatment (Table 3). There was no impact in either group in the H&Y evaluation.

In the NMS, the rTMS group showed detriment in mood/cognition (one point of difference) and gastrointestinal tract (three points of difference). In the miscellaneous group, the general evaluation showed a benefit (nine points of difference) because of a positive impact on weight change (two points of difference) and excessive sweating (four points of difference). The sham group experienced detriment in cardiovascular assessment (three points) and urinary symptoms (four points). However, the evaluation showed improvements in sleep/fatigue symptoms (10 points), attention and memory (1 point), sexual function (4.5 points of difference), and miscellaneous (9 points of difference) (Table 3).

The rTMS and sham groups showed differences in the DRS without reaching statistical significance. In both groups, dyskinesia worsened in historical and objective assessments, with the sham group being the most affected (Table 2).

No adverse effects were detected during and one week after the last brain stimulation.

## 4. Discussion

We investigated the clinical effects of the long-term application of HF-rTMS over M1 on PD with LID, evaluating the effects on motor and non-motor PD symptomatology and LIDs.

The results from the MDS-UPDRS assessments for the sham group indicated improvements in Parts 1 to 3 compared to baseline evaluations; however, these changes lacked statistical significance. In the rTMS group, improvements were observed in Parts 1 and 2 without statistical significance. Conversely, deterioration was noted in Parts 3 and 4, with Part 3 showing statistically significant deterioration. A detailed analysis of the MDS-UPDRS Part 3 revealed that this decline was associated with increased symptom severity on the left side, particularly in hand movement and kinetic tremor, which demonstrated a statistically significant difference, as well as in posture. No significant changes were observed in the Hoehn and Yahr Scale for either group. The assessment of motor symptoms in the rTMS group exhibited modifications in Part 3, the objective evaluation conducted by the neurologist.

Additionally, our laboratory’s findings and other studies indicate that HF-rTMS effectively improves Parkinsonian motor symptoms, mainly when applied bilaterally over the motor cortex [12,31,32,33,34,35,36]. Different studies have improved the UPDRS-III score from 19% to 49% depending on stimulation frequency (5–20 Hz) over the motor cortex [30,37,38,39]. To date, bilateral stimulation with HF-rTMS on M1 can be recommended for the treatment of motor Parkinsonian symptoms. The long-term effect has been contradictory [40,41]. Our results showed that long-term HF-rTMS did not modify motor symptomatology in PD-LID patients, allowing a progression rate consistent with the expected.

Regarding non-motor symptoms, the sham group exhibited a general improvement, particularly in sleep/fatigue and other miscellaneous symptoms; however, these changes did not reach statistical significance. In contrast, the rTMS group experienced a decline in mood/cognition and gastrointestinal parameters, which also lacked statistical significance, and the corresponding subanalyses failed to elucidate the underlying causes of this deterioration. rTMS has been applied to the prefrontal cortex in patients with PD for the treatment of depression. Studies indicate that HF-rTMS is not superior to sham treatments or selective serotonin reuptake inhibitors. Notably, the combination of rTMS with antidepressants may demonstrate greater efficacy than antidepressants alone [12,31]. Our findings should be interpreted with the understanding that we stimulated M1, which would not inherently impact mood or cognition. This study did not include scales for measuring mood, which would have allowed for an objective assessment of these changes; additionally, in the domain of cognition, the Mini-Mental State Examination was applied only at baseline as an inclusion criterion. The Dyskinesia Rating Scale showed progression in both groups, with a statistical trend in the rTMS group’s objective evaluation of the right arm and drinking. Previous studies investigating the effects of HF-rTMS in patients with PD-LIDs differ in sample size (ranging from four to eight participants), dyskinesia measurement scales used (Abnormal Involuntary Movement Scale and the Unified Parkinson’s Disease Rating Scale), the frequency of stimulation (one to five sessions), and HF-rTMS protocol. None of these studies assessed the long-term effects of HF-rTMS [40,42,43,44]. The present study, with a more robust sample and a more precise dyskinesia evaluation, concurs with these prior findings, demonstrating no significant impact of HF-rTMS on LIDs.

The HF-rTMS protocol in this study was derived from a previous trial that reported positive outcomes over motor symptom control in PD, where 40% of the cohort presented LID [31]. Our results did not replicate the previously reported improvements in motor symptoms, suggesting that the effects observed in earlier studies may not extend to a population solely comprised of PD-LID patients.

We expected three positive outcomes: improved motor symptom control that could allow levodopa dose adjustment, direct control over LID, or a combination of both. However, our findings do not support any long-term therapeutic benefit of HF-rTMS for motor symptoms in PD-LID patients nor indicate a direct ameliorative effect on LIDs.

Despite the inherent limitations of our pilot study, particularly concerning sample size, it is essential to emphasize the therapeutic adherence and favorable safety profile of long-term HF-rTMS application.

## Figures and Tables

**Table 1:** Clinical characteristics description of the eleven PD patients. No statistical differences were observed between the rTMS and sham groups.

**Population Characteristics**		**rTMS**	**Sham**	*p*
**Number of patients**		9	2	
**Age (years; median; 25–75 centile)**		73(65.5–77)	53.5 (53–54)	0.098
**Sex distribution (%)**	Female	3 (33.33%)	1 (50%)	0.658
	Male	6 (66.66%)	1 (50%)	
**Laterality (%)**	Right	9 (100%)	2 (100%)	-
	Left	0	0	
**Scholarity (years; median; 25–75 centile)**		6(4.5–9)	8 (7–9)	0.329
**Age at diagnosis of PD (years; median; 25–75 centile)**		62(52.5–68.5)	43 (39–47)	0.098
**Years with PD (years; median; 25–75 centile)**		10 (5.5–15)	10.5 (7–14)	0.813
**Initial affected site (%)**	Right	3 (33.33%)	0	0.887
	Left	6 (66.66%)	2 (100%)	
**Years with levodopa treatment (years; median; 25–75 centile)**		7(4.5–13)	5 (0)	0.469
**Actual levodopa dose (mg/day; median; 25–75 centile)**		800 (762.5–1212.5)	875 (700–1050)	0.477
**Actual dopa agonist treatment (%)**	Yes	7 (77.77%)	2 (100%)	0.461
	No	2 (22.22%)	0	
**Actual amantadine treatment (%)**	Yes	3 (33.33%)	1 (50%)	0.658
	No	6 (66.66%)	1 (50%)	

**Table 2:** Baseline and post-stimulation clinical characteristics. p1 compares the first and last evaluations in the stimulated arm, analyzed with the Wilcoxon test. p2 compares the first and last evaluations in the sham group, analyzed with the Wilcoxon test. p3 compares the initial assessment between both groups, analyzed with the U Mann–Whitney test. p4 compares the last evaluation between both groups, analyzed with the U Mann–Whitney test. H&Y—Hoehn and Yahr Scale; NMS—Non-Motor Symptom Assessment Scale for PD; UDysRS—Unified Dyskinesia Rating Scale.

Clinical Evaluation		rTMS (n = 9)		p1	Sham (n = 2)		p2	p3	p4
		First Evaluation (Centile 25–75)	Last Evaluation (Centile 25–75)		First Evaluation (Range)	Last Evaluation (Range)			
**MDS-UPDRS**	Part 1 (median)	16(6.5–20)	14(8.5–17)	0.859	18 (16–20)	13.5 (5–22)	0.655	0.55	0.813
	Part 2 (median)	19 (8.5–24.5)	14 (11.5–23)	0.888	21(8–34	19 (11–28)	0.655	0.723	0.906
	Part 3 (median)	34 (27.5–37)	40 (33–44.5)	**0.021**	55(43–67)	42 (30–55)	0.18	**0.059**	0.813
	Part 4 (median)	4 (1.5–6.5)	7 (3–8.5)	0.153	6.5(1–12)	7 (1–13)	0.317	0.812	1
**H&Y**	2 (total; %)	5 (55.55%)	6 (66.66%)	0.317	1 (50%)	1 (50%)	0.317	0.509	0.673
	3 (total; %)	4 (44.44%)	3 (33.33)		0	1 (50%)			
	4 (total, %)	0	0		1 (50%)	0			
**NMS**	Cardiovascular (median)	1 (0–3)	0 (0- 0.5)	0.48	5 (0–10)	8	0.317	0.62	0.294
	Sleep/fatigue (median)	8 (3.5–21)	5 (2–14)	0.179	27.5 (27–28)	17(5–29)	0.655	0.099	0.406
	Mood/cognition (median)	0 (0–7.5)	1 (0–11)	**0.066**	10.5 (9–12)	9 (0–18)	0.655	0.23	0.805
	Perceptual problems/hallucinations (median)	0 (0–4)	0 (0–2.5)	0.891	1 (0–2)	0 (0)	0.317	0.898	0.275
	Attention/memory(median)	3 (0–13)	3 (0–8.5)	1	1.5(1–2)	0.5 (0–1)	0.157	0.72	0.227
	Gastrointestinal tract (median)	6 (5–13)	8(5.5–19.5)	**0.066**	5 (1–9)	6 (0–12)	0.655	0.72	0.476
	Urinary (median)	18 (3–20.5)	12 (4.5–32)	0.44	12 (7–17)	16 (0–32)	0.655	0.637	0.721
	Sexual function (median)	1 (0–15)	0 (0–6.5)	0.109	4.5 (0–9)	0 (0)	0.317	0.805	0.368
	Miscellaneous (median)	13 (5.5–16.5)	4 (0–4)	**0.018**	13 (12–14)	4 (0–8)	0.18	1	0.612
	Total score (median)	79 (31.5–91.5)	52 (14.5–102)	0.26	80 (72–88)	60.5 (5–116)	0.655	0.722	0.814
**UDysRS**	Historical (median)	14 (3.23.5)	15 (12.5–26)	0.312	15.5 (7–24)	24 (10–38)	0.18	0.55	0.814
	Objective (median)	7(3–8.5)	9 (8–16.5)	0.074	6 (5–7)	12.5 (7–18)	0.18	1	0.905
	Total (median)	21(7–31.5)	25 (20.5–42)	0.213	21.5 (12–31)	36.5 (17–56)	0.18	0.637	0.814

**Table 3:** Analysis of the items showed statistical differences obtained after stimulation. *p* compares the first and last evaluations of the stimulated arm, analyzed by the Wilcoxon test. NMS-Non-Motor Symptom Assessment Scale for PD; DRS-Dyskinesia Rating Scale (DRS).

Clinical Evaluation			rTMS (n = 9)		
			First Evaluation (Centile 25–75)	Last Evaluation (Centile 25–75)	*p*
**MDS-UPDRS**	Part 3 (median)	General (median; centile 25–72)	34 (27.5–37)	40 (33–44.5)	**0.021**
		Right-hand movement	2 (1–2)	2 (2–2)	0.083
		Left-hand movement	2 (1–2)	3 (2–3)	**0.053**
		Right pronation/supination	1 (1–1)	1 (1–1.5)	0.083
		Right leg agility	1 (0.5–2)	1 (1–2)	0.083
		Posture	1 (1–2)	2 (1.5–2)	**0.059**
		Left-hand postural tremor	0 (0)	0 (0–1)	0.083
		Left-hand kinetic tremor	0 (0–0.5)	1 (1–1)	**0.02**
**NMS**	Mood/cognition (median)	General (median; centile 25–72)	0 (0–7.5)	1 (0–11)	0.066
		Sad or depressed	0 (0–3)	0 (0–5)	0.066
	Gastrointestinal tract (median)		6 (5–13)	8 (5.5–19.5)	0.066
	Miscellaneous (median) ^a^	General (median; centile 25–72)	13 (5.5–16.5)	4 (0–4)	**0.018**
		Recent change in weight	2(0–4)	0(0)	**0.038**
		Excessive sweating	4 (0–12)	0 (0–2.5)	**0.041**

^a^: Integrated by pain, taste/smell, change in weight, and excessive sweating.

## Data Availability

The original contributions presented in the study are included in the article; further inquiries can be directed to the corresponding authors.

## References

[1] Martinez-Ramirez D., Rodriguez-Violante M., Velazquez-Avila E.S., Cervantes-Arriaga A., Gonzalez-Cantu A., Corona T., Velasquez-Perez L. (2020). Incidence and geographic distribution of Parkinson’s disease in Mexico. Salud Publica Mex..

[2] Kulisevsky J. (2022). Pharmacological management of Parkinson’s disease motor symptoms: Update and recommendations from an expert. Rev. Neurol..

[3] Cervantes-Arriaga A., Rodríguez-Violante M., López-Ruiz M., Estrada-Bellmann I., Zuñiga-Ramírez C., Otero-Cerdeira E., Camacho-Ordoñez A., González-Latapi P., Morales-Briceño H., Martínez-Ramírez D. (2013). Profile characterization of Parkinson’s disease in Mexico: ReMePARK study. Gac. Med. Mex..

[4] Guridi J., Gonzalez-Redondo R., Obeso J.A. (2012). Clinical features, pathophysiology, and treatment of levodopa-induced dyskinesias in Parkinson’s disease. Parkinsons Dis..

[5] Wu J., Lim E.C., Nadkarni N.V., Tan E.K., Kumar P.M. (2019). The impact of levodopa therapy-induced complications on quality of life in Parkinson’s disease patients in Singapore. Sci. Rep..

[6] Hansen C.A., Miller D.R., Annarumma S., Rusch C.T., Ramirez-Zamora A., Khoshbouei H. (2022). Levodopa-induced dyskinesia: A historical review of Parkinson’s disease, dopamine, and modern advancements in research and treatment. J. Neurol..

[7] Deuschl G., Schade-Brittinger C., Krack P., Volkmann J., Schafer H., Botzel K., Daniels C., Deutschlander A., Dillmann U., Eisner W. (2006). A randomized trial of deep-brain stimulation for Parkinson’s disease. N. Engl. J. Med..

[8] Espay A.J., Morgante F., Merola A., Fasano A., Marsili L., Fox S.H., Bezard E., Picconi B., Calabresi P., Lang A.E. (2018). Levodopa-induced dyskinesia in Parkinson disease: Current and evolving concepts. Ann. Neurol..

[9] Ramirez-Zamora A., Ostrem J.L. (2018). Globus Pallidus Interna or Subthalamic Nucleus Deep Brain Stimulation for Parkinson Disease: A Review. JAMA Neurol..

[10] Fan S.Y., Wang K.L., Hu W., Eisinger R.S., Han A., Han C.L., Wang Q., Michitomo S., Zhang J.G., Wang F. (2020). Pallidal versus subthalamic nucleus deep brain stimulation for levodopa-induced dyskinesia. Ann. Clin. Transl. Neurol..

[11] Fitzgerald P.B., Fountain S., Daskalakis Z.J. (2006). A comprehensive review of the effects of rTMS on motor cortical excitability and inhibition. Clin. Neurophysiol..

[12] Lefaucheur J.P., Andre-Obadia N., Antal A., Ayache S.S., Baeken C., Benninger D.H., Cantello R.M., Cincotta M., de Carvalho M., De Ridder D. (2014). Evidence-based guidelines on the therapeutic use of repetitive transcranial magnetic stimulation (rTMS). Clin. Neurophysiol..

[13] Lefaucheur J.P. (2019). Transcranial magnetic stimulation. Handb. Clin. Neurol..

[14] Kricheldorff J., Goke K., Kiebs M., Kasten F.H., Herrmann C.S., Witt K., Hurlemann R. (2022). Evidence of Neuroplastic Changes after Transcranial Magnetic, Electric, and Deep Brain Stimulation. Brain Sci..

[15] Hallett M. (2007). Transcranial magnetic stimulation: A primer. Neuron.

[16] Rothwell J.C., Hallett M., Berardelli A., Eisen A., Rossini P., Paulus W. (1999). Magnetic stimulation: Motor evoked potentials. The International Federation of Clinical Neurophysiology. Electroencephalogr. Clin. Neurophysiol. Suppl..

[17] Pascual-Leone A., Valls-Sole J., Wassermann E.M., Hallett M. (1994). Responses to rapid-rate transcranial magnetic stimulation of the human motor cortex. Brain.

[18] Ziemann U. (2004). TMS induced plasticity in human cortex. Rev. Neurosci..

[19] Ziemann U., Paulus W., Nitsche M.A., Pascual-Leone A., Byblow W.D., Berardelli A., Siebner H.R., Classen J., Cohen L.G., Rothwell J.C. (2008). Consensus: Motor cortex plasticity protocols. Brain Stimul..

[20] Siebner H.R., Bergmann T.O., Bestmann S., Massimini M., Johansen-Berg H., Mochizuki H., Bohning D.E., Boorman E.D., Groppa S., Miniussi C. (2009). Consensus paper: Combining transcranial stimulation with neuroimaging. Brain Stimul..

[21] Eldaief M.C., Halko M.A., Buckner R.L., Pascual-Leone A. (2011). Transcranial magnetic stimulation modulates the brain’s intrinsic activity in a frequency-dependent manner. Proc. Natl. Acad. Sci. USA.

[22] Wang J.X., Rogers L.M., Gross E.Z., Ryals A.J., Dokucu M.E., Brandstatt K.L., Hermiller M.S., Voss J.L. (2014). Targeted enhancement of cortical-hippocampal brain networks and associative memory. Science.

[23] Raij T., Nummenmaa A., Marin M.F., Porter D., Furtak S., Setsompop K., Milad M.R. (2018). Prefrontal Cortex Stimulation Enhances Fear Extinction Memory in Humans. Biol. Psychiatry.

[24] Ziemann U., Hallett M., Cohen L.G. (1998). Mechanisms of deafferentation-induced plasticity in human motor cortex. J. Neurosci..

[25] Cirillo G., Di Pino G., Capone F., Ranieri F., Florio L., Todisco V., Tedeschi G., Funke K., Di Lazzaro V. (2017). Neurobiological after-effects of non-invasive brain stimulation. Brain Stimul..

[26] Touge T., Gerschlager W., Brown P., Rothwell J.C. (2001). Are the after-effects of low-frequency rTMS on motor cortex excitability due to changes in the efficacy of cortical synapses?. Clin. Neurophysiol..

[27] Peinemann A., Reimer B., Loer C., Quartarone A., Munchau A., Conrad B., Siebner H.R. (2004). Long-lasting increase in corticospinal excitability after 1800 pulses of subthreshold 5 Hz repetitive TMS to the primary motor cortex. Clin. Neurophysiol..

[28] Rothkegel H., Sommer M., Paulus W. (2010). Breaks during 5Hz rTMS are essential for facilitatory after effects. Clin. Neurophysiol..

[29] Lefaucheur J.P., Aleman A., Baeken C., Benninger D.H., Brunelin J., Di Lazzaro V., Filipovic S.R., Grefkes C., Hasan A., Hummel F.C. (2020). Evidence-based guidelines on the therapeutic use of repetitive transcranial magnetic stimulation (rTMS): An update (2014–2018). Clin. Neurophysiol..

[30] Gonzalez-Garcia N., Armony J.L., Soto J., Trejo D., Alegria M.A., Drucker-Colin R. (2011). Effects of rTMS on Parkinson’s disease: A longitudinal fMRI study. J. Neurol..

[31] Chou Y.H., Hickey P.T., Sundman M., Song A.W., Chen N.K. (2015). Effects of repetitive transcranial magnetic stimulation on motor symptoms in Parkinson disease: A systematic review and meta-analysis. JAMA Neurol..

[32] Kim M.S., Chang W.H., Cho J.W., Youn J., Kim Y.K., Kim S.W., Kim Y.H. (2015). Efficacy of cumulative high-frequency rTMS on freezing of gait in Parkinson’s disease. Restor. Neurol. Neurosci..

[33] Zanjani A., Zakzanis K.K., Daskalakis Z.J., Chen R. (2015). Repetitive transcranial magnetic stimulation of the primary motor cortex in the treatment of motor signs in Parkinson’s disease: A quantitative review of the literature. Mov. Disord..

[34] Brys M., Fox M.D., Agarwal S., Biagioni M., Dacpano G., Kumar P., Pirraglia E., Chen R., Wu A., Fernandez H. (2016). Multifocal repetitive TMS for motor and mood symptoms of Parkinson disease: A randomized trial. Neurology.

[35] Makkos A., Pal E., Aschermann Z., Janszky J., Balazs E., Takacs K., Karadi K., Komoly S., Kovacs N. (2016). High-Frequency Repetitive Transcranial Magnetic Stimulation Can Improve Depression in Parkinson’s Disease: A Randomized, Double-Blind, Placebo-Controlled Study. Neuropsychobiology.

[36] Yang C., Guo Z., Peng H., Xing G., Chen H., McClure M.A., He B., He L., Du F., Xiong L. (2018). Repetitive transcranial magnetic stimulation therapy for motor recovery in Parkinson’s disease: A Meta-analysis. Brain Behav..

[37] Khedr E.M., Farweez H.M., Islam H. (2003). Therapeutic effect of repetitive transcranial magnetic stimulation on motor function in Parkinson’s disease patients. Eur. J. Neurol..

[38] Khedr E.M., Rothwell J.C., Shawky O.A., Ahmed M.A., Hamdy A. (2006). Effect of daily repetitive transcranial magnetic stimulation on motor performance in Parkinson’s disease. Mov. Disord..

[39] Maruo T., Hosomi K., Shimokawa T., Kishima H., Oshino S., Morris S., Kageyama Y., Yokoe M., Yoshimine T., Saitoh Y. (2013). High-frequency repetitive transcranial magnetic stimulation over the primary foot motor area in Parkinson’s disease. Brain Stimul..

[40] Koch G., Brusa L., Caltagirone C., Peppe A., Oliveri M., Stanzione P., Centonze D. (2005). rTMS of supplementary motor area modulates therapy-induced dyskinesias in Parkinson disease. Neurology.

[41] Brusa L., Versace V., Koch G., Iani C., Stanzione P., Bernardi G., Centonze D. (2006). Low frequency rTMS of the SMA transiently ameliorates peak-dose LID in Parkinson’s disease. Clin. Neurophysiol..

[42] Rektorova I., Sedlackova S., Telecka S., Hlubocky A., Rektor I. (2008). Dorsolateral prefrontal cortex: A possible target for modulating dyskinesias in Parkinson’s disease by repetitive transcranial magnetic stimulation. Int. J. Biomed. Imaging.

[43] Cerasa A., Koch G., Donzuso G., Mangone G., Morelli M., Brusa L., Stampanoni Bassi M., Ponzo V., Picazio S., Passamonti L. (2015). A network centred on the inferior frontal cortex is critically involved in levodopa-induced dyskinesias. Brain.

[44] Wu Y., Cao X.B., Zeng W.Q., Zhai H., Zhang X.Q., Yang X.M., Cheng C., Wang J.L., Yang X.M., Xu Y. (2021). Transcranial Magnetic Stimulation Alleviates Levodopa-Induced Dyskinesia in Parkinson’s Disease and the Related Mechanisms: A Mini-Review. Front. Neurol..

